# Integrated geoelectrical and geological data sets for shallow structure characterization of the southern margin of the Krzeszowice Graben (Southern Poland)

**DOI:** 10.1016/j.dib.2019.104157

**Published:** 2019-06-20

**Authors:** Tomasz Woźniak, Grzegorz Bania

**Affiliations:** Faculty of Geology, Geophysics and Environment Protection, AGH University of Science and Technology, Al. A. Mickiewicza 30, 30-059 Kraków, Poland

**Keywords:** Electrical resistivity tomography, Microfacies analysis, Upper Jurassic limestones, Krzeszowice Graben

## Abstract

The data presented in this paper are related to the characterization of the selected part of the southern margin of the Krzeszowice Graben in Southern Poland. The presented data article contains geoelectrical and geological data. The first of them include: three (3) field profiles of 2D electrical resistivity tomography (ERT) and also three (3) 2D ERT resistivity models. The second data set encompasses selected fourteen (14) photomicrographs of thin sections from undertaken Upper Jurrasic limestones samples. ERT field data was acquired with the use of SuperSting R8 resistivity meter, while the 2D resistivity models were generated in Res2Dmod software. Both geoelectrical data sets have been processed using Res2Dinv software. From all collected limestones samples thin sections have been prepared and studied for microfacies analysis.

**Table:** Specifications table

Subject area	*Geophysics and geology*
More specific subject area	*Geoelectrical resistivity and sedimentary geology*
Type of data	*Figure, Table, KML, XLS, STG, DAT, IVP, INV, MOD, JPG files*
How data was acquired	• Electrical resistivity tomography (ERT) using SuperSting™ Wi-Fi (R8 – eight channel) • Geoelectrical forward 2D ERT modeling (Res2Dmod software) • Geological field work (sampling procedure) • OLYMPUS SZX10 microscope (thin section analysis) and Dunham textural classification scheme with its later modifications
Data format	*Raw, processed, inverted*
Experimental factors	*The field apparent resistivity along with calculated (based on resistivity models) apparent resistivity data sets were inverted and together with geological data were analysed in order to characterise geological features.*
Experimental features	*Geoelectrical survey involving 2D Electrical resistivity tomography (ERT), forward 2D resistivity modeling and geological survey (sampling procedure) have been conducted.*
Data source location	*Nawojowa Góra (Winnica Hill), Krzeszowice Graben, Southern Poland*
Data accessibility	*All the data sets are available with this article.*
Related research article	*T. Woźniak, G. Bania, Analysis of the tectonic and sedimentary features of the southern margin of the Krzeszowice Graben in Southern Poland based on an integrated geoelectrical and geological studies, J. Appl. Geophys., 165, 2019, 60–76* [1]

**Table:** 

**Value of the data** • The data presents how integrated methods (both geophysical and geological) can be used for characterisation of the tectonic features and subsurface lithology units of a selected part of the Krzeszowice Graben in southern Poland. • Resistivity modeling data can be used in order to broaden potential geological interpretation. • The classified rocks could be compared to other Upper Jurassic sediments deposited on the shelf bordered a vast, epicontinental sea that rimmed the Tethys Ocean from the north. • The datasets may give the scientific basis and pave the way to the selected future directions of the research for studies of that area. • The data sets can be used for educational purposes.

## 1 Data

**Fig. 1:**
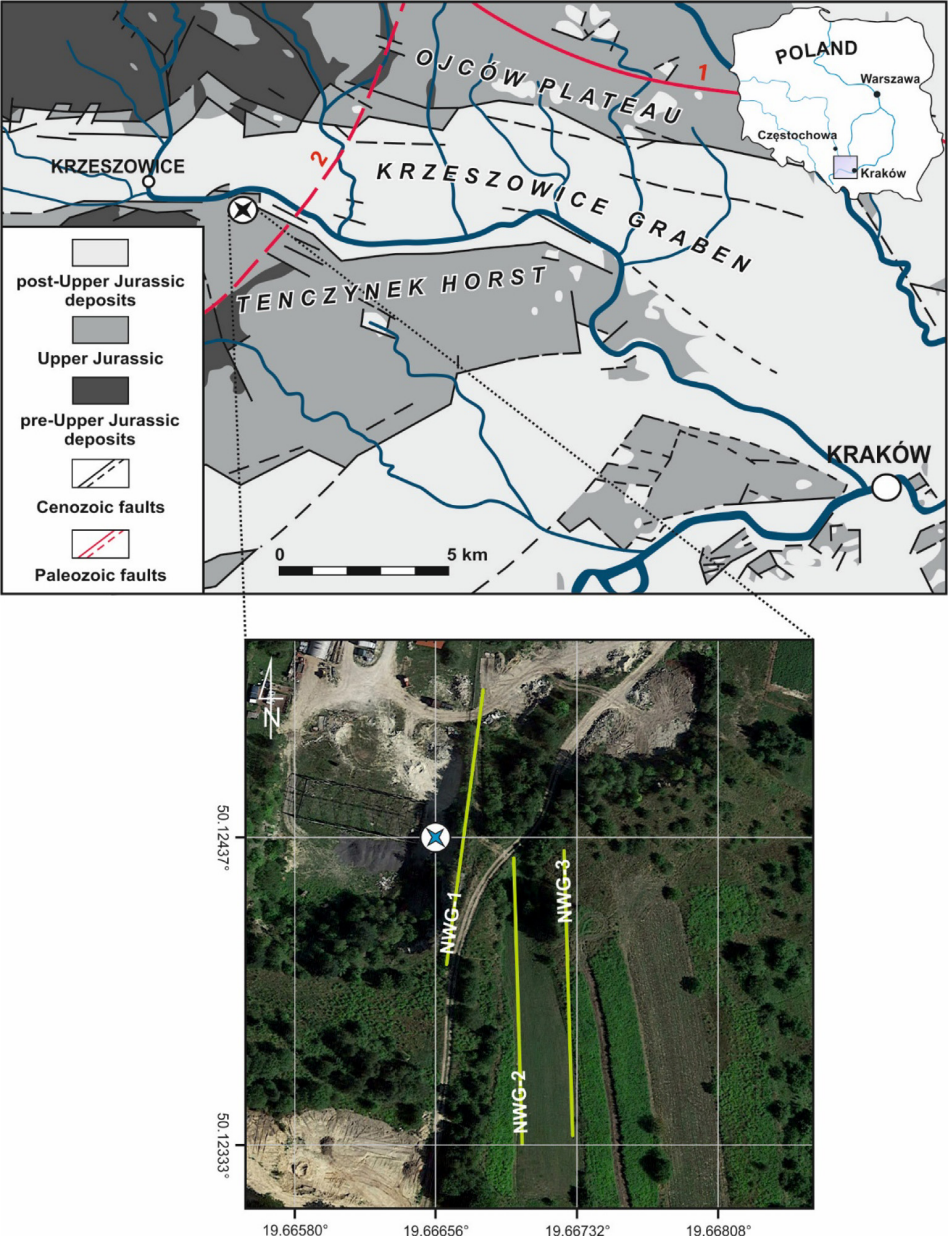
Maps showing: (upper part) detailed location of the research area (black asterisk) superimposed on geological map of the southern part of the Kraków-Częstochowa Upland (geology after [7]; simplified and modified) with positions of Kraków-Lubliniec Fault Zone (1) and Krzeszowice-Charsznica Fault (2) within the homocline basement (location after [8]); (lower part) digital orthophotomap showing the locations of three ERT survey lines (NWG 1-3) and sampling site (blue asterisk).

Herein, the data sets constitute the basis for the research article entitled “Analysis of the tectonic and sedimentary features of the southern margin of the Krzeszowice Graben in Southern Poland based on an integrated geoelectrical and geological studies” by Woźniak and Bania, 2019 [1]. The attached to this paper data includes: (i) Google Earth KML file with the location of ERT profiles and geological sampling area; (ii) XLS file with ERT electrodes elevations (in local height system); (iii) XLS file with “a” and “n” parameters for dipole-dipole (DD) and Wenner-Schlumberger (WS) arrays used to collect field and forward 2D modeling data; (iv) SuperSting STG files with raw ERT data collected in the field; (v) Raw and processed ERT field data DAT files together with synthetic ERT data calculated during forward 2D modeling DAT files (both data sets can be used for inversion in Res2Dinv software); (vi) Res2Dinv IVP files which contains all inversion parameters used by authors in inversion process; (vii) Res2Dmod MOD files with 2D resistivity models that refer to field data; (viii) Res2Dinv INV files with the inversion results of field and synthetic data sets; (ix) selected thin-section photomicrographs (JPG files) of examined Upper Jurassic limestones. Presented in this paper data numbered from (i) to (viii) are available in Appendix A, whereas the latter, numbered as (ix) are contained in Appendix B. Detailed location of the sampling site is listed in Table 1 and shown in Fig. 1 (blue asterisk).

**Table 1:** GPS coordinates of the ERT profiles and the geological sampling location.

ERT profiles	Latitude	Longitude	Location
NWG–1	50.124877°	19.666809°	beginning
	50.123915°	19.666640°	end
NWG–2	50.124290°	19.666982°	beginning
	50.123295°	19.667030°	end
NWG–3	50.124319°	19.667233°	beginning
	50.123322°	19.667288°	end
geological sampling	50.124370°	19.666560°	–

## 2 Experimental design, materials and methods

### 2.1 Geology of the research area

The Krzeszowice Graben, positioned within the southern part of the Kraków-Częstochowa Upland (Southern Poland), is one of the biggest tectonic structure of the regional tectonic unit of Silesian-Kraków Homocline (Fig. 1). The homocline, composed of Mesozoic sedimentary rocks extends on the NW-SE direction and dips gently, at the low angle, towards NE. Differentiated Mesozoic formations of the homocline overlie unconformably the folded Precambrian and Palaeozoic basement that is disturbed by Kraków-Lubliniec Fault Zone and Krzeszowice-Charsznica Fault (1 and 2 in Fig. 1). It is assumed that Krzeszowice Graben is a structure and product of extensional tectonics which began to affect in Cenozoic and has been generated by the overthrusting Outer Carpathian nappes. It is worth noting that some of the faults that frame the Krzeszowice Graben might have been formed already in Late Jurassic or even earlier and they have been later rejuvenated [e.g. [2], and references therein]. The Krzeszowice Graben is limited respectively, by the Tenczynek Horst to the south and the Ojców Plateau to the north (Fig. 1). From a geological point of view, the predominant role in the geological structure of the graben margins is played by the Upper Jurassic carbonates of variable facies development [3]. They encompass three individual facies such as massive, bedded as well as peculiar basinal facies covering the submarine gravity flows deposits [4]. The latter ones, in particular, occur along the graben margins [5], [6]. Marine Miocene clays constitues a graben filling.

### 2.2 Data acquisition

The preliminary geological survey encompassing exposure recording and sampling procedure in the research site (Nawojowa Góra quarry) was undertaken previously to the geophysical survey. Standard oriented thin sections for observation with transmitted light microscopy were prepared from limestones samples, which have been spanning the entire thickness of the Upper Jurassic limestones at the lower bench of the quarry (cf. [1], and Fig. 1).

The geoelectrical field survey consists of three ERT profiles which have been performed on 14 July 2017 in the area placed nearby abandoned quarry in Nawojowa Góra (Fig. 1). Profiles (NWG–1, NWG–2 and NWG–3) each about 110 m long, run on the approximately N–S direction. The geographical coordinates of the beginning and end of each profile are presented in Table 1. SuperSting R8 resistivity meter with the set of 56 electrodes has been applied. The electrodes spacing in each profile was 2 m. Data sets have been acquired with dipole-dipole and Wenner-Schlumberger arrays. The collected data for these arrays were then combined into one for each survey line.

The resistivity models were generated through forward 2D ERT modeling using the finite-element method with the application of the Res2Dmod software (ver. 3.02.01). The models were supposed to reflect the field data, so the following parameters were used: 56 electrodes, basic electrode spacing 2 m, the same topographical data as for the appropriate field ERT profiles. For simplicity, calculated apparent resistivity pseudosections have been acquired only with the use of dipole-dipole array with the same ‘a’ and ‘n’ parameters as applied in the field measurements.

### 2.3 Data processing

The microscopic data, obtained with OLYMPUS SZX10 microscope, have been interpreted using the Dunham [9] textural classification scheme with its later modification.

The measurement data for dipole-dipole and Wenner-Schlumberger arrays obtained during the ERT field survey have been combined. Then, the data was processed in Res2Dinv software (ver. 3.59.119). The ‘RMS error statistics' option has been applied. It allowed discarding some bad points at cutoff error level 20% for all field data sets. The inversion was conducted with two, developed by authors, slightly different sets of parameter settings available in the program. The difference is the use of two different variants: robust (L_1_-norm) and smoothness-constrained (L_2_-norm) methods [10]. Another program settings are the same for both authors inversion parameter sets. Some of these are: ‘Reduce effect of side blocks’ with ‘Slight’ setting; the same width of inversion mesh blocks; 2 mesh nodes between adjacent electrodes.

Inversion of the synthetic data sets, obtained from the 2D resistivity models have been carried out with the use of Res2Dinv software and with authors ‘robust’ inversion parameters set.
