# Supplementary figures and images for: Integrated geoelectrical and geological data sets for shallow structure characterization of the southern margin of the Krzeszowice Graben (Southern Poland)
Source: Data Brief. 2019 Jun 20;25:104157. doi: 10.1016/j.dib.2019.104157 (PMC6624450; doi:10.1016/j.dib.2019.104157)

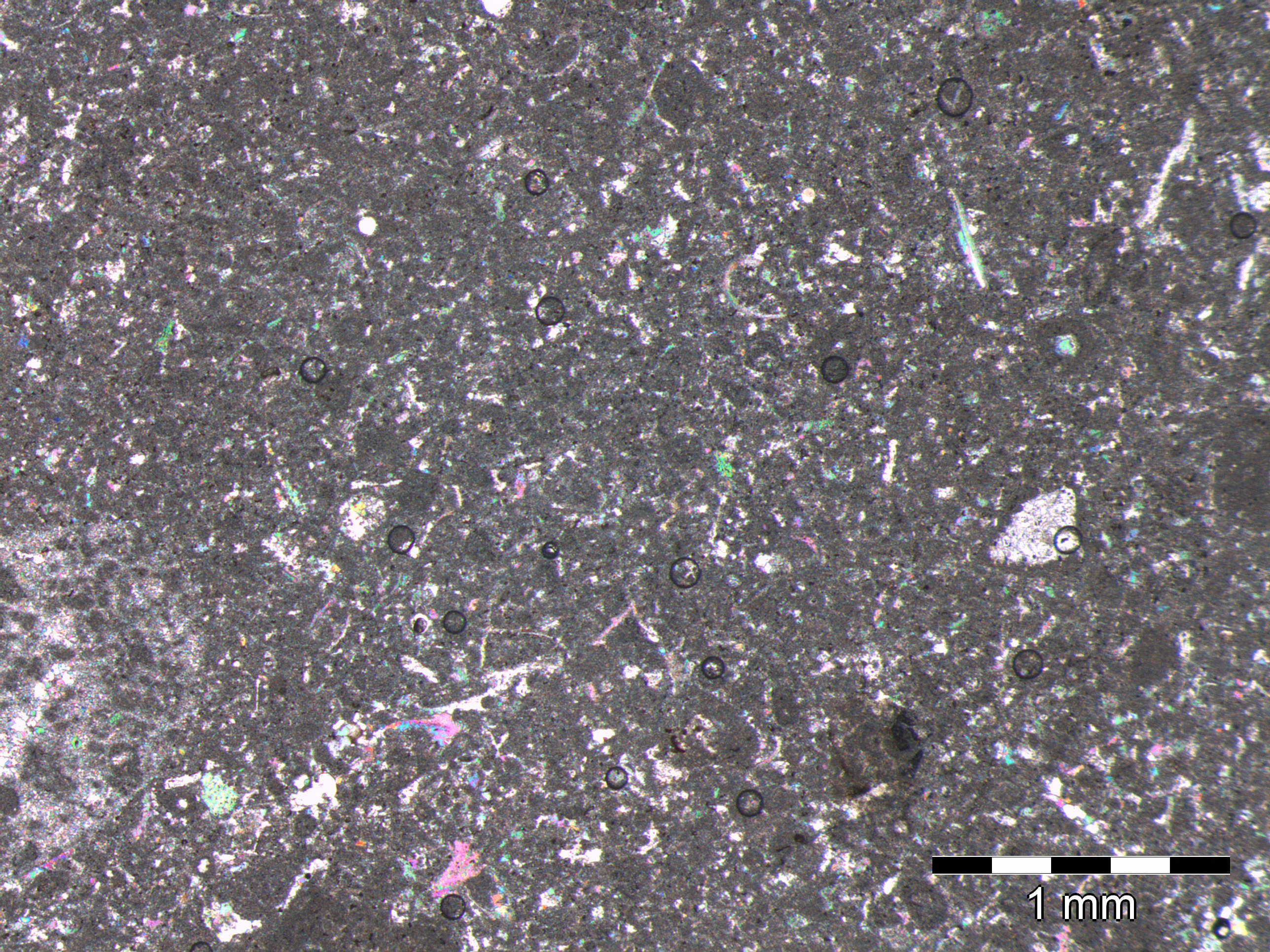

Supplement: Supplementary file 1 [file mmc1.zip › Appendix B/NWG1-2.jpg]

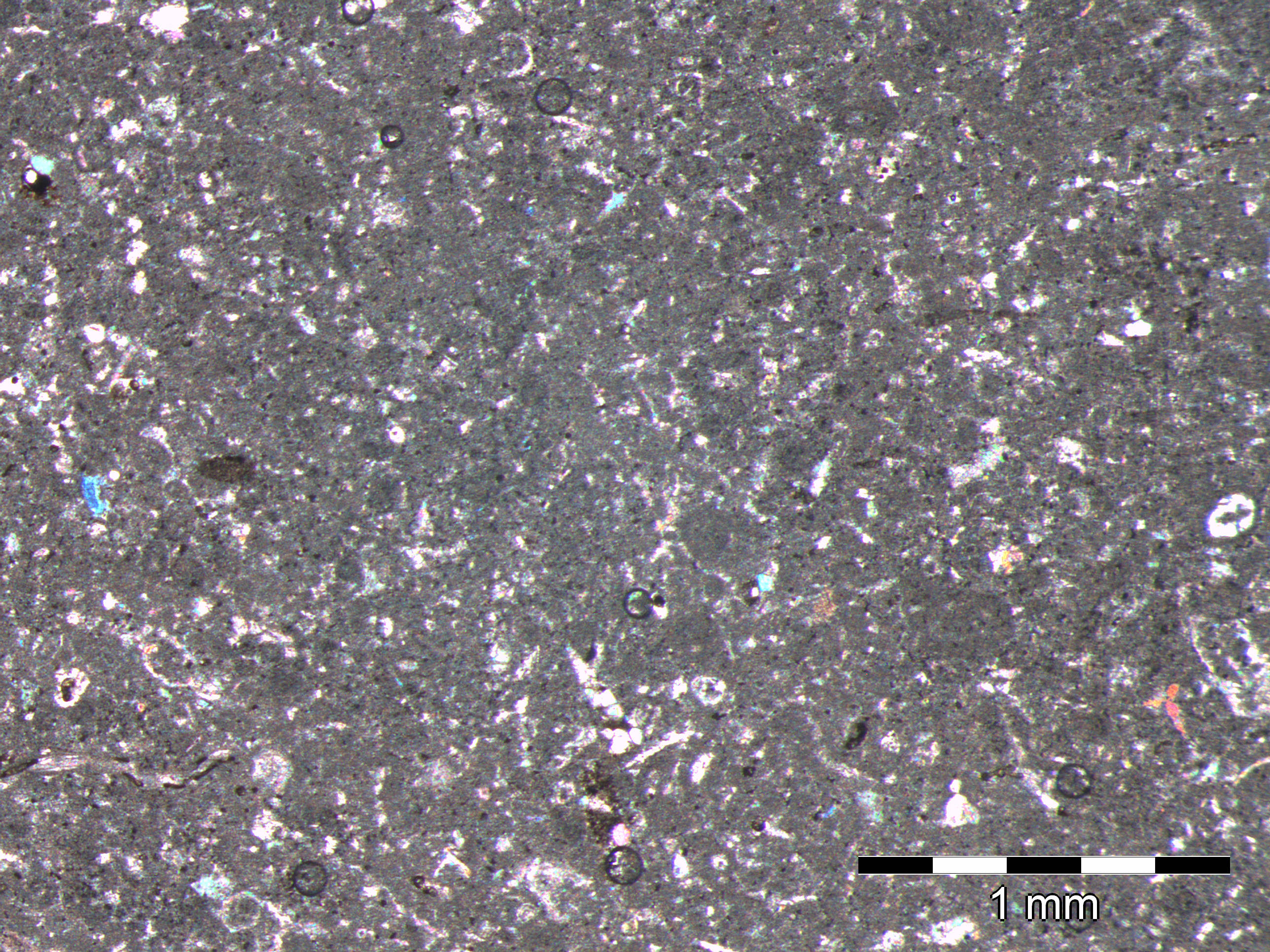

Supplement: Supplementary file 1 [file mmc1.zip › Appendix B/NWG1.jpg]

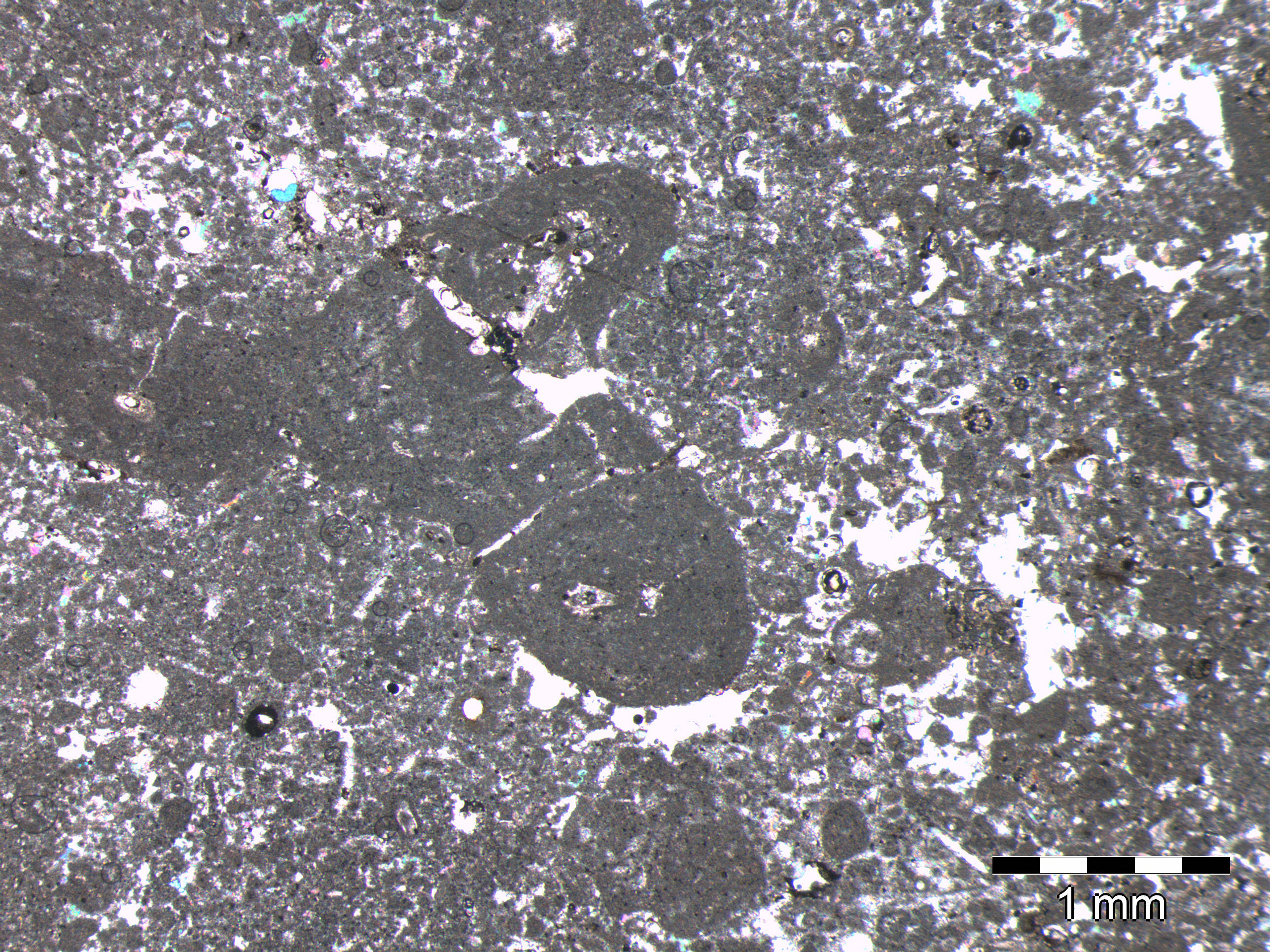

Supplement: Supplementary file 1 [file mmc1.zip › Appendix B/NWG2Ad-1.jpg]

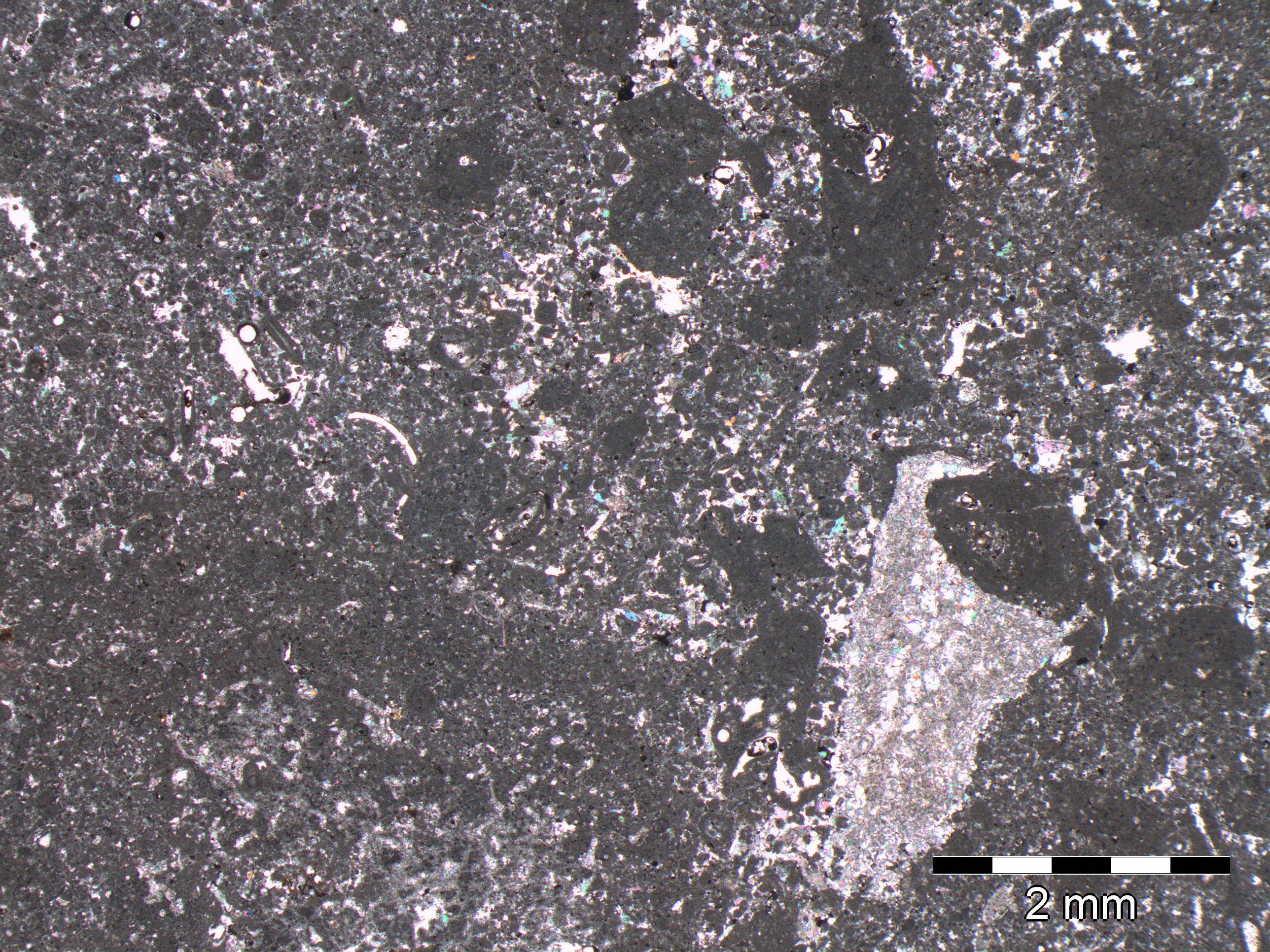

Supplement: Supplementary file 1 [file mmc1.zip › Appendix B/NWG2Ad-2.jpg]

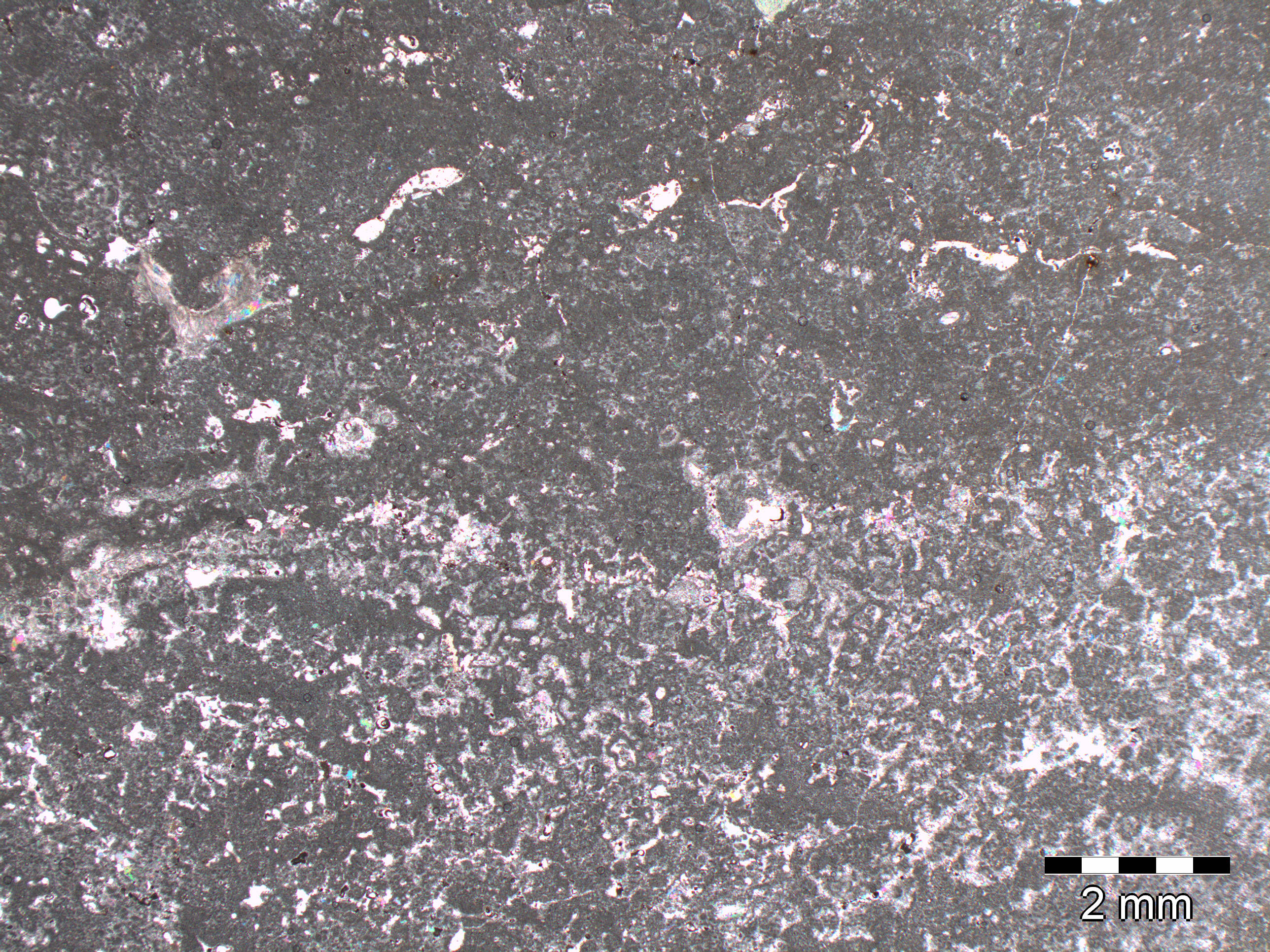

Supplement: Supplementary file 1 [file mmc1.zip › Appendix B/NWG2Ag.jpg]

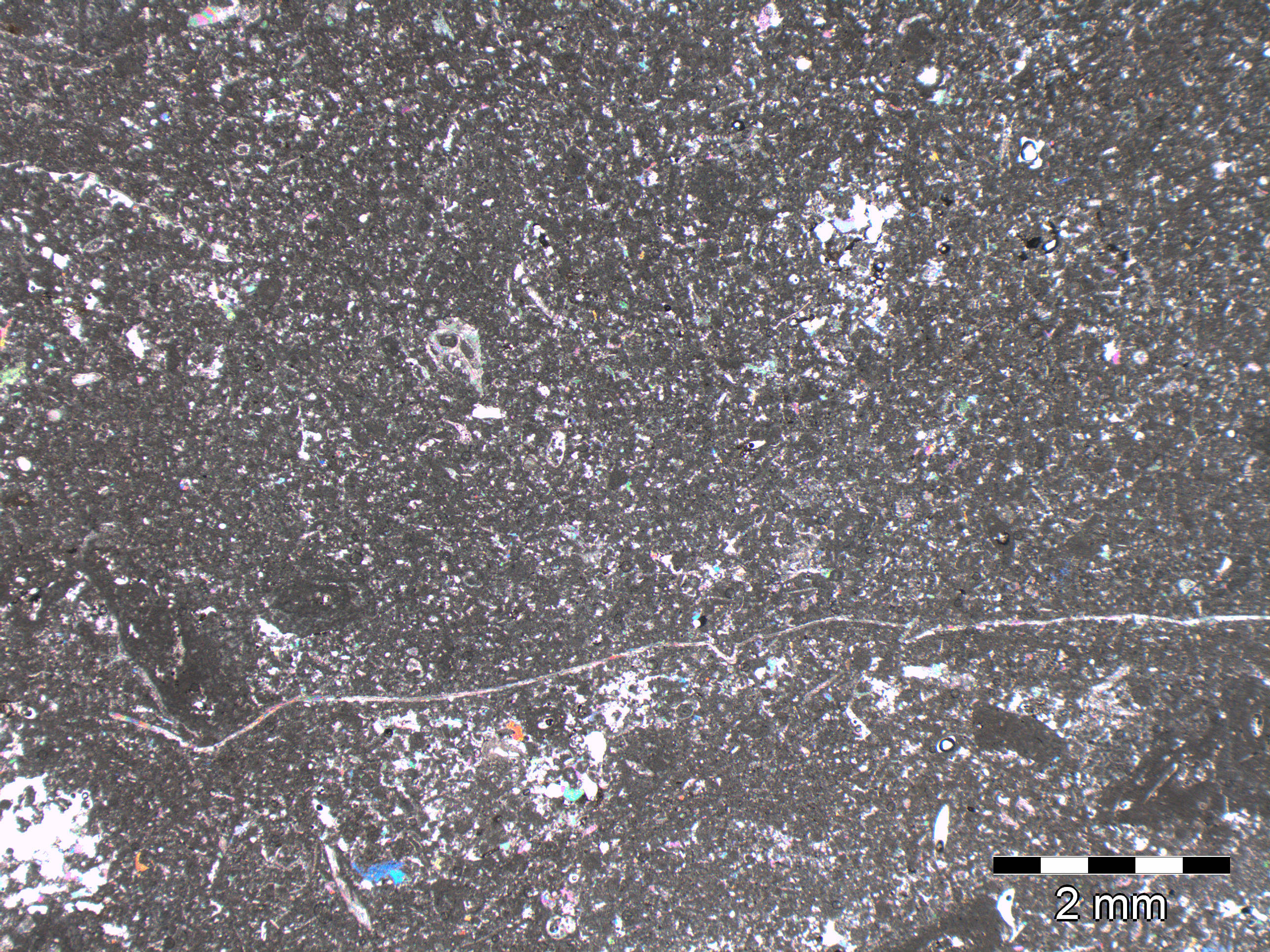

Supplement: Supplementary file 1 [file mmc1.zip › Appendix B/NWG2Bd-1.jpg]

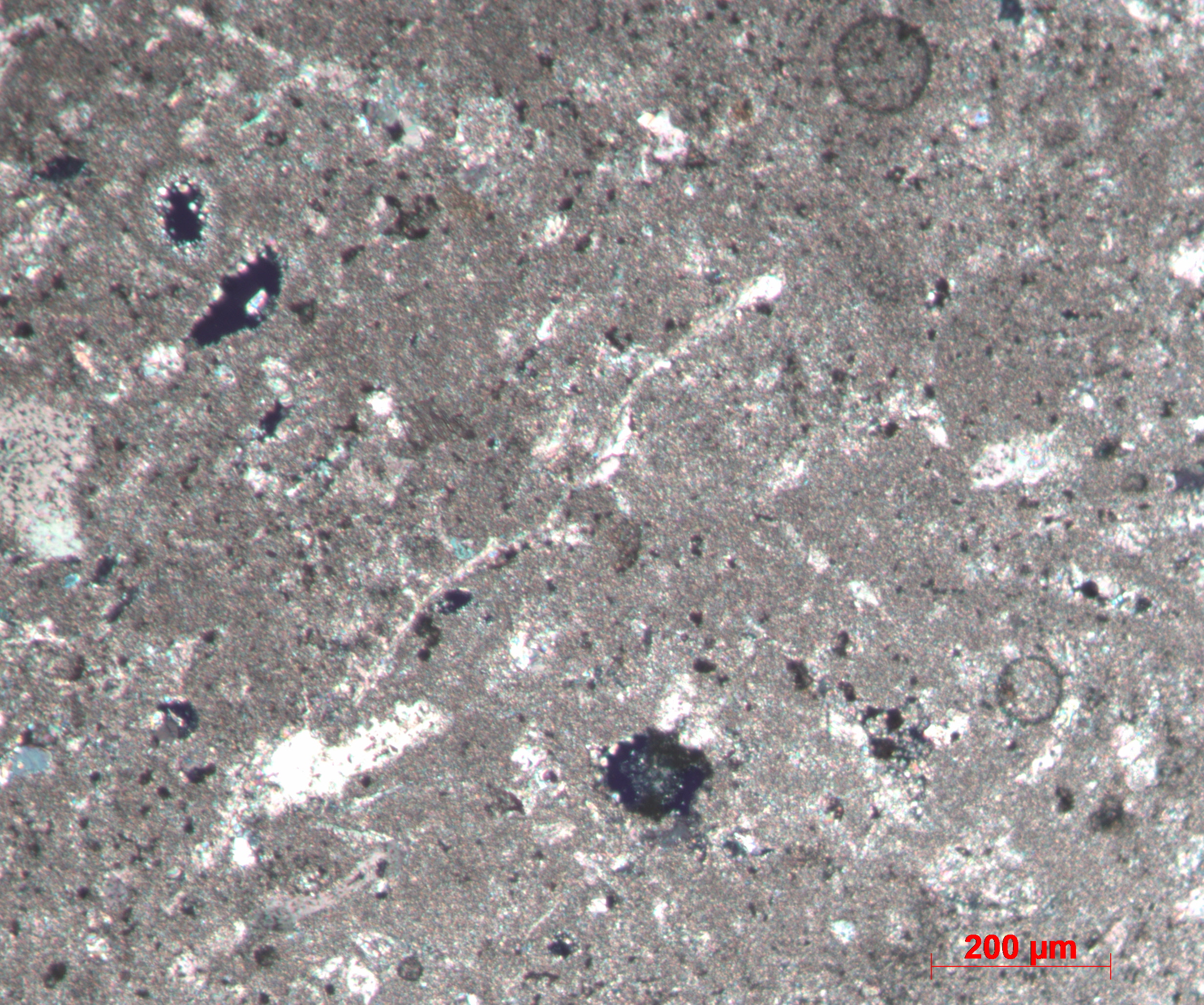

Supplement: Supplementary file 1 [file mmc1.zip › Appendix B/NWG2C_1d.jpg]

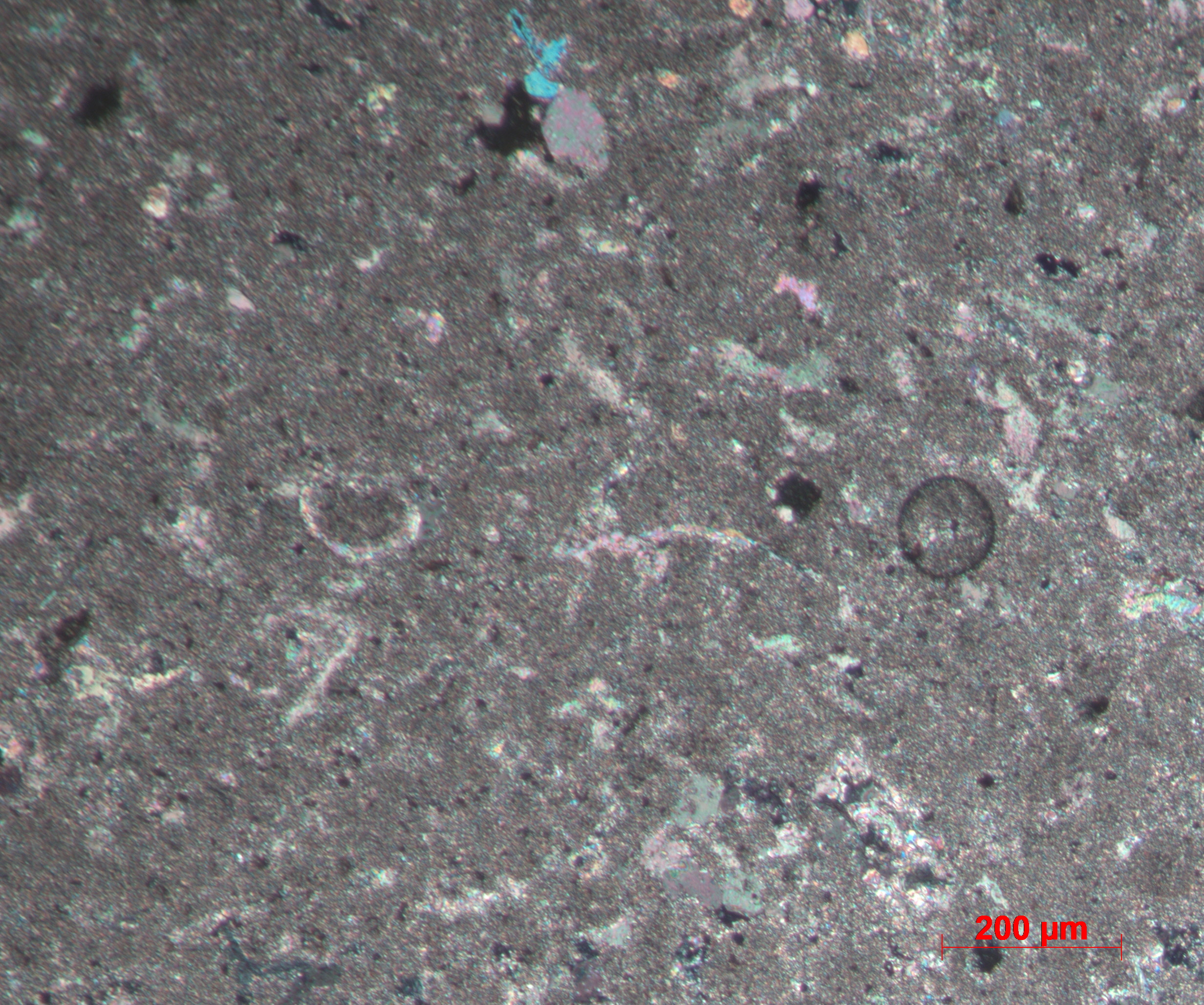

Supplement: Supplementary file 1 [file mmc1.zip › Appendix B/NWG2dod.jpg]

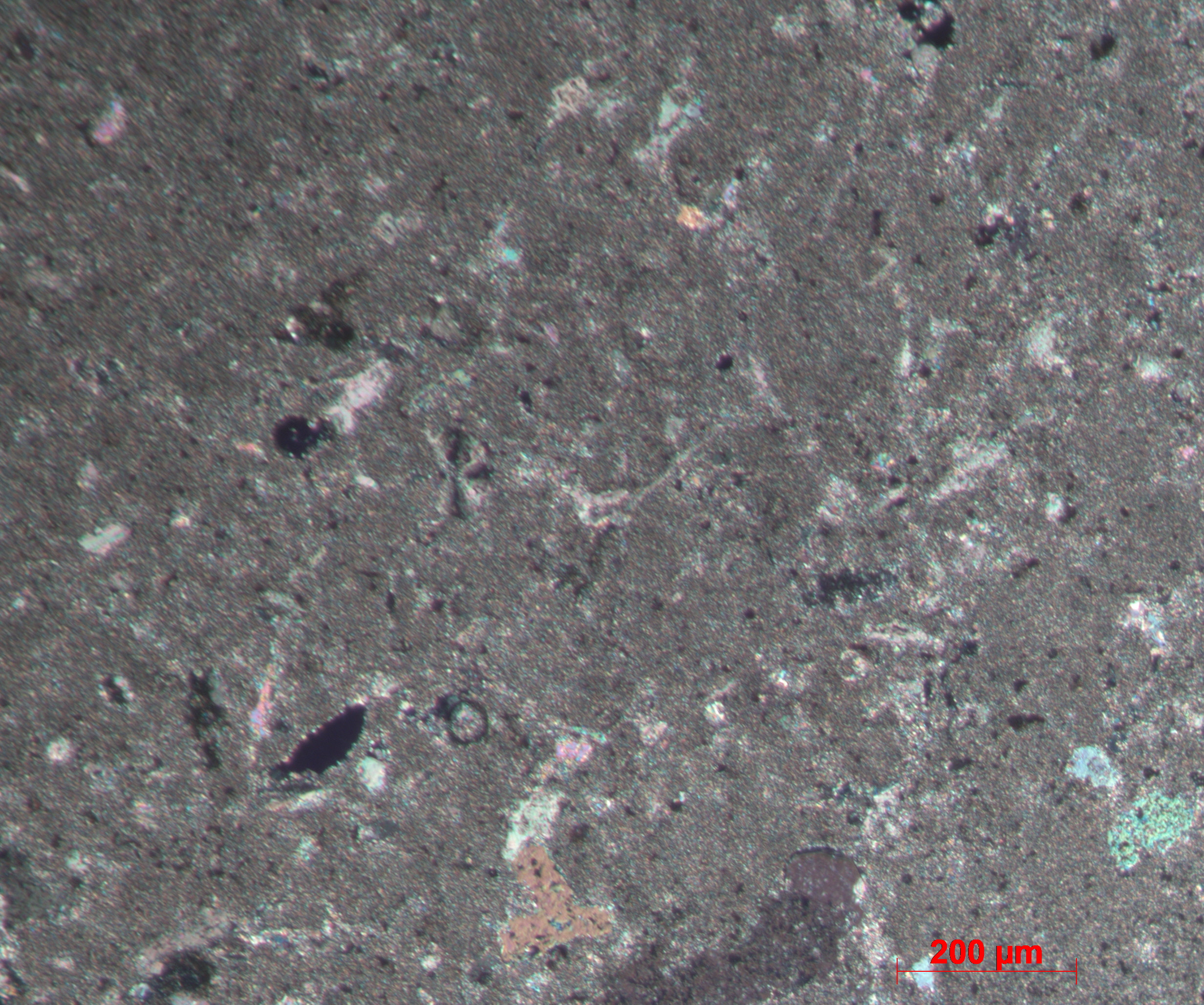

Supplement: Supplementary file 1 [file mmc1.zip › Appendix B/NWG2dod1.jpg]

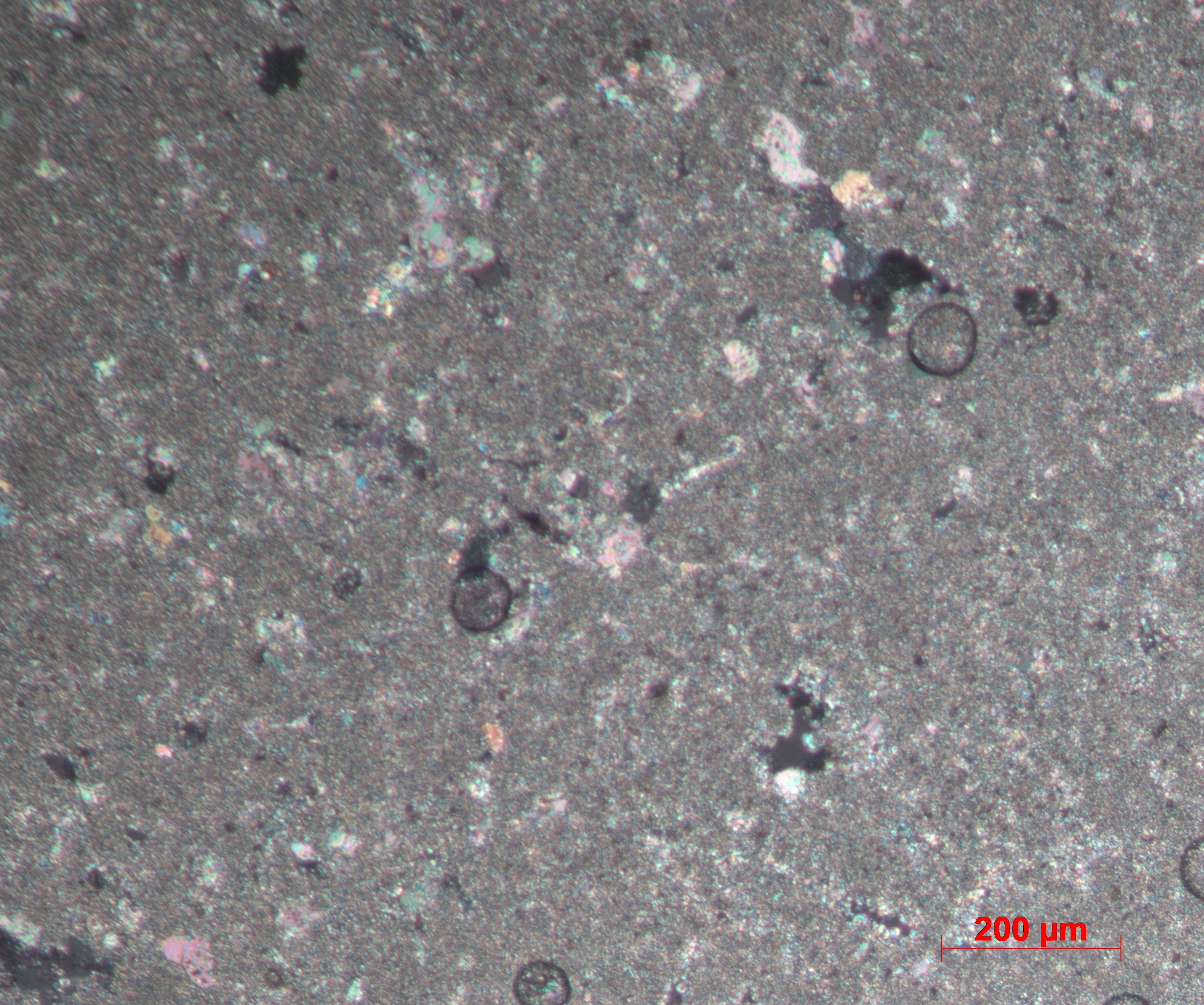

Supplement: Supplementary file 1 [file mmc1.zip › Appendix B/NWG2dod2.jpg]

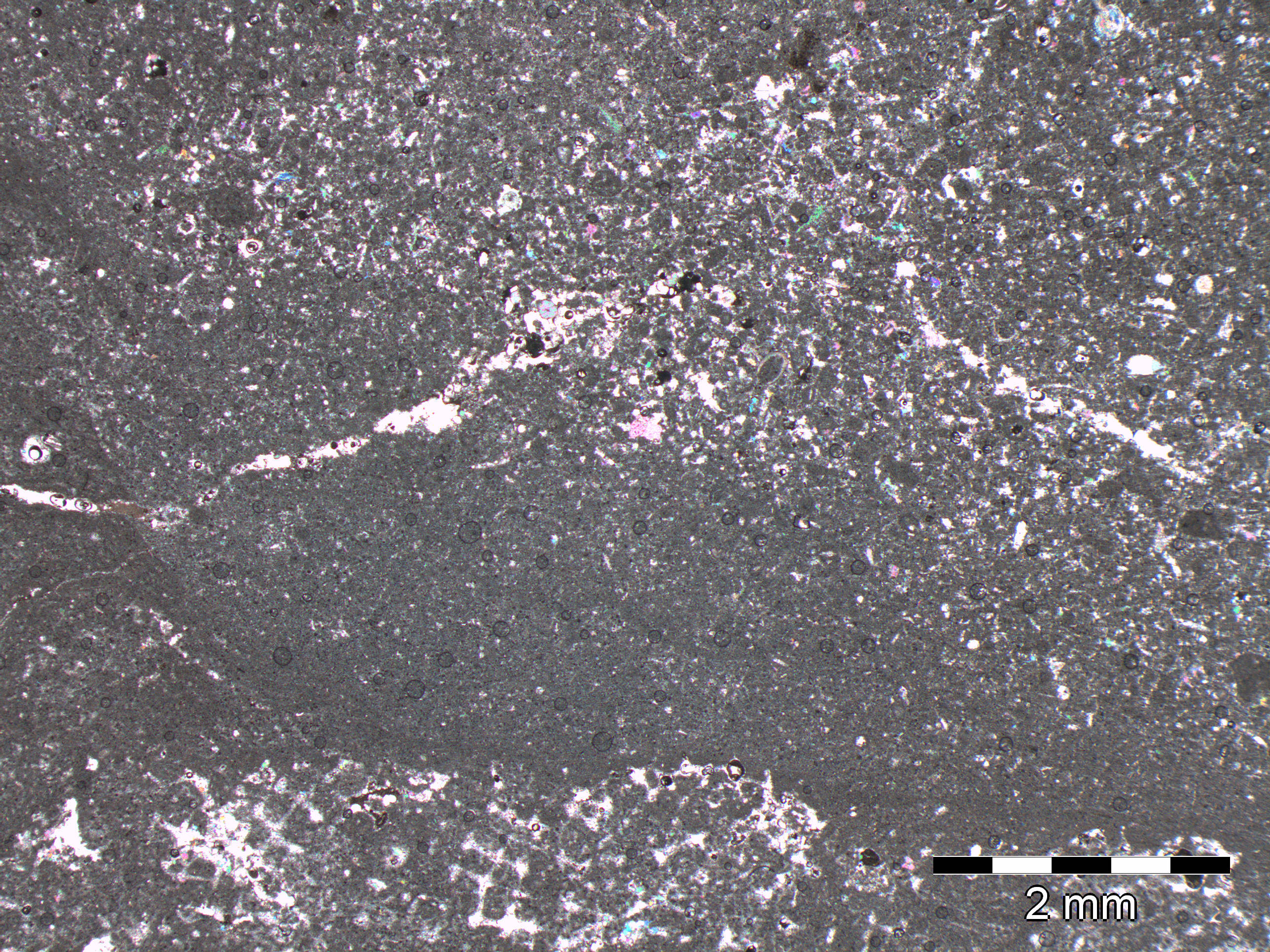

Supplement: Supplementary file 1 [file mmc1.zip › Appendix B/NWG4-1.jpg]

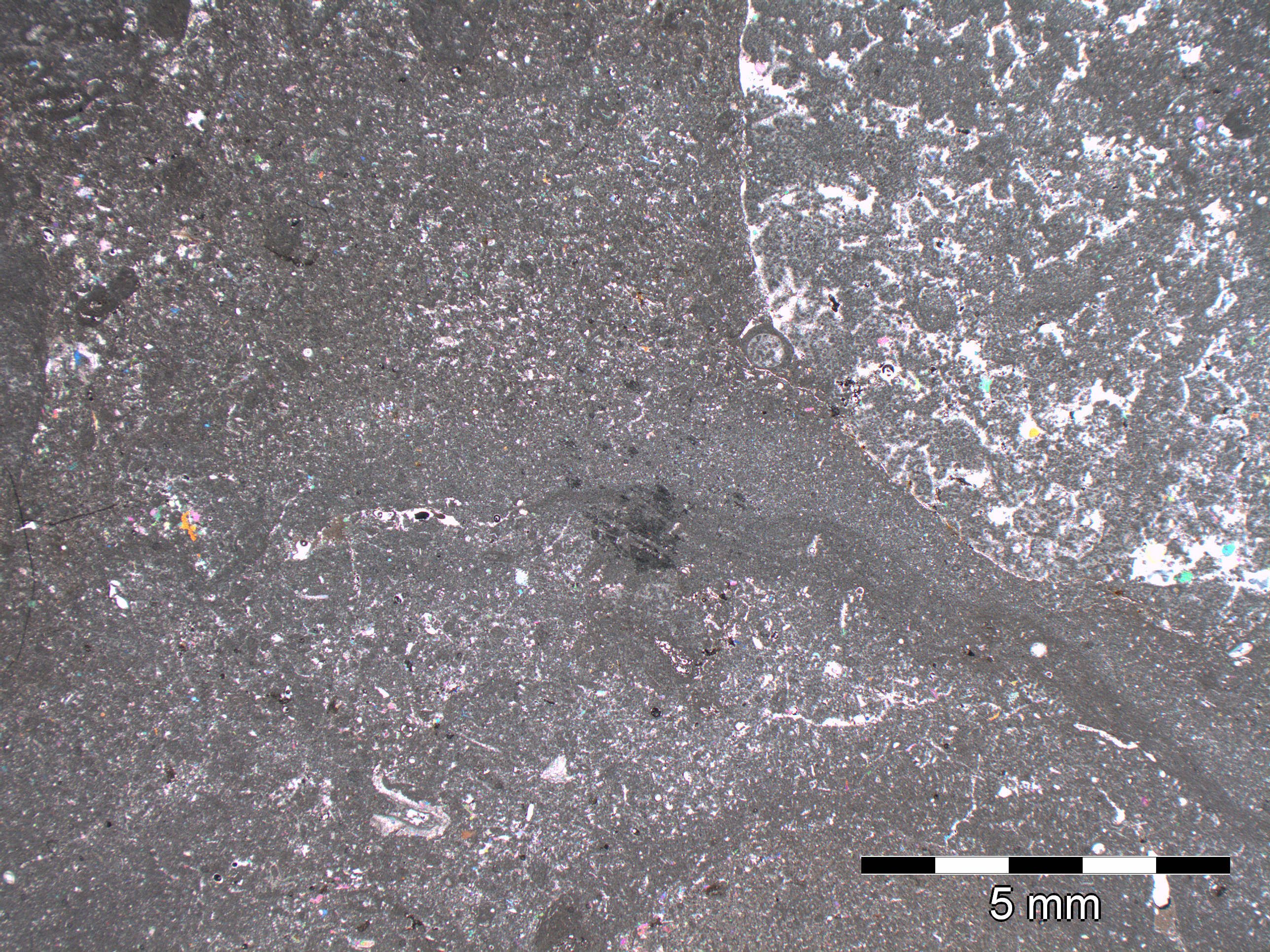

Supplement: Supplementary file 1 [file mmc1.zip › Appendix B/NWG4-3.jpg]

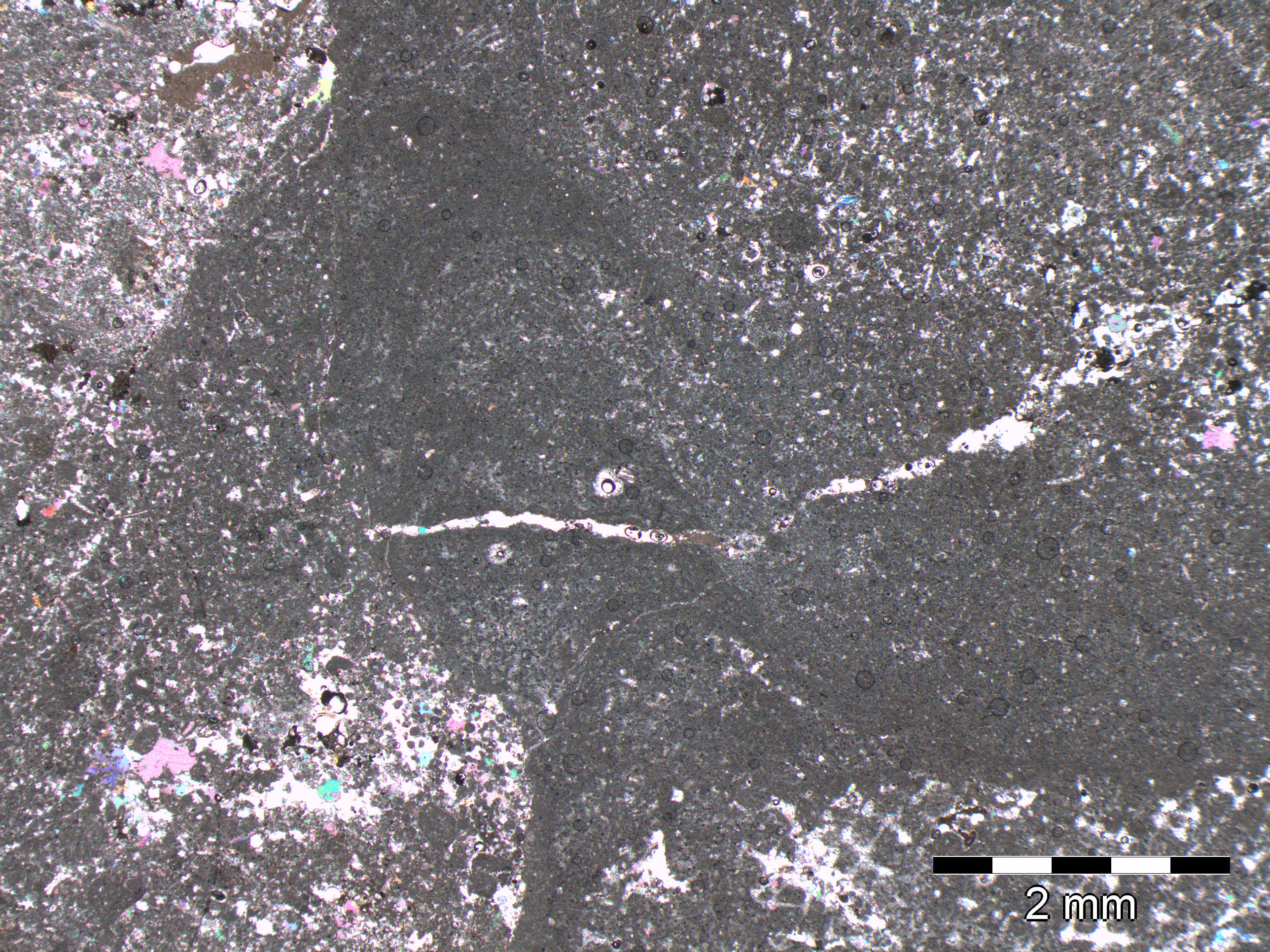

Supplement: Supplementary file 1 [file mmc1.zip › Appendix B/NWG4.jpg]

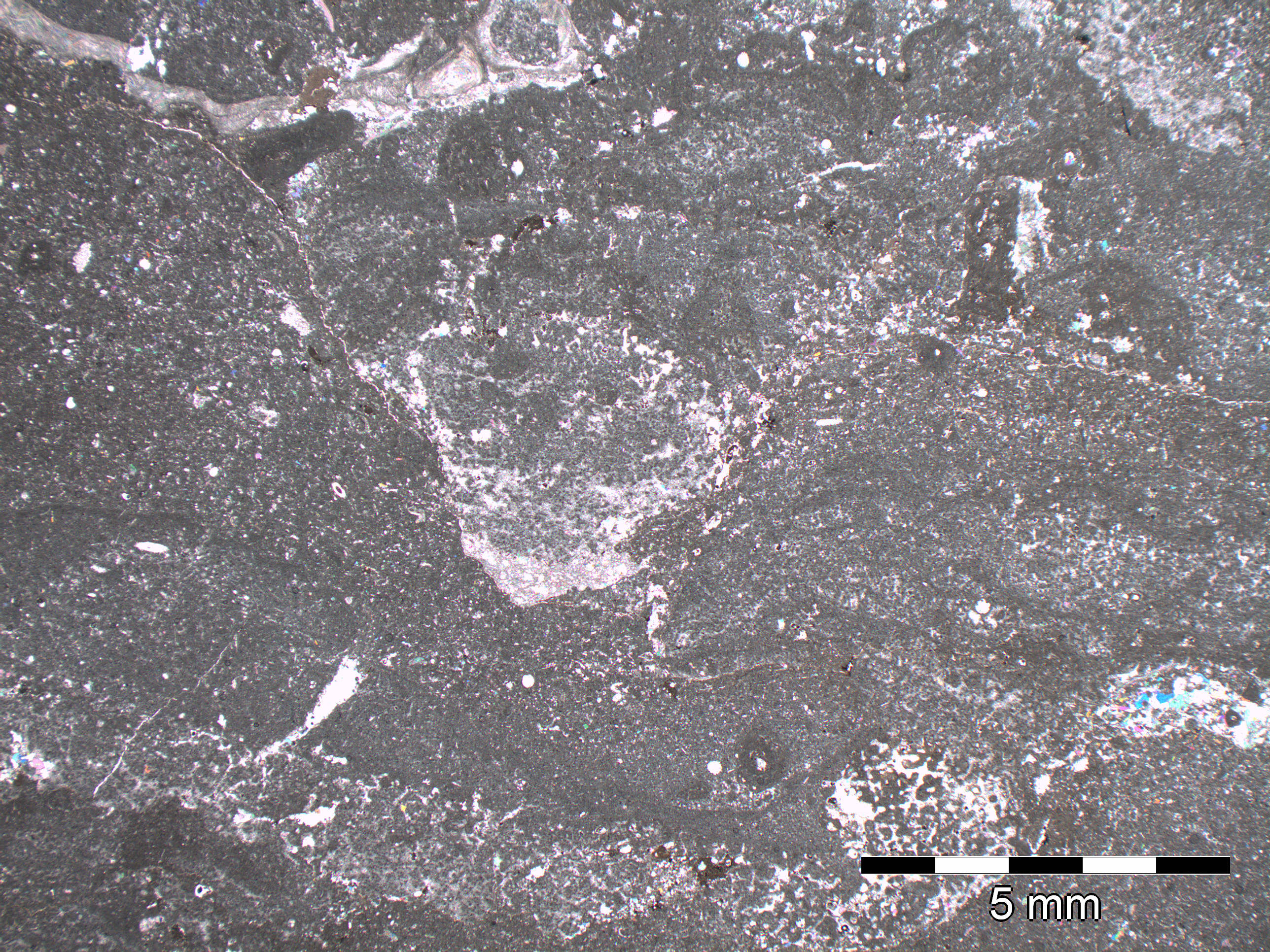

Supplement: Supplementary file 1 [file mmc1.zip › Appendix B/NWG5.jpg]
